# Prediction of maximum oxygen uptake over time in adults: analysis from the FRIEND registry

**DOI:** 10.1590/1414-431X2025e13731

**Published:** 2025-03-03

**Authors:** V.Z. Dourado, A.C. Barbosa, M.S.M.P. Simões, V.T. Lauria, A.C. Matheus, K.P. Sadarangani, R.L. Arantes, M. Romiti, J.E. Peterman, R. Arena, M.P. Harber, J. Myers, L.A. Kaminsky

**Affiliations:** 1 Departamento de Ciências do Movimento Humano, Universidade Federal de São Paulo, Santos, SP, Brasil; 2 Lown Scholars in Cardiovascular Health Program, Harvard T.H. Chan School of Public Health, Boston, MA, USA; 3 Universidad Autónoma de Chile, Providencia, Santiago, Chile; 4 Escuela de Kinesiología, Facultad de Salud y Odontología, Universidad Diego Portales, Santiago, Chile; 5 Instituto de Medicina Cardiovascular Angiocorpore, Santos, SP, Brasil; 6 Clinical Exercise Physiology, Ball State University, Muncie, IN, USA; 7 Department of Physical Therapy, College of Applied Science, University of Illinois Chicago, Chicago, IL, USA; 8 Health Sciences, Taylor University, Upland, IN, USA; 9 Division of Cardiology, Veterans Affairs Palo Alto Healthcare System and Stanford University, Palo Alto, CA, USA

**Keywords:** *V̇*O_2max_, Cardiorespiratory fitness, Cardiopulmonary exercise testing, Cardiovascular risk

## Abstract

Maximum oxygen uptake (*V̇*O_2max_) equations from developed countries are inaccurate for developing countries. Accordingly, we aimed to develop equations to predict treadmill *V̇*O_2max_ over time based on variables other than exercise test in adults from the USA and Brazil undergoing cardiopulmonary exercise testing (CPET). We analyzed data from 2,170 adults who underwent two CPETs (1,307 men; 20-85 years) from the USA (n=1,880) and Brazil (n=290) with a second test after 2.0±1.7 years on average. We fit linear mixed-effects models to develop equations using 90% of the sample, randomly selected. In the remaining 10% of the cohort, we used the coefficient of variation, intraclass correlation coefficient, and the Bland and Altman plots to cross-validate the optimal equation. Our best linear mixed model equation was as follows: *V̇*O_2max_ (mLO_2_·kg^-1^·min^-1^) = 62.01 - (0.23×Age_years_) - (0.001×Age×Age) - (0.65×Body mass index_kg/m_^2^) + (5.47×Sex_females=0; males=1_) + (2.78×Country_Brazil=0; USA=1_) - (0.68×Arterial hypertension_no=0; yes=1_) - (0.45×Hyperlipidemia_no=0; yes=1_) - (2.02×Smoking_no=0; yes=1_) - (4.36×Insufficiently active_no=0; yes=1_) - (1.67×Beta-blockers_no=0; yes=1_); R^2^=0.566. Our main equation was reliable at baseline according to Bland and Altman plot results (mean difference, 0.01 mLO_2_·kg^-1^·min^-1^: 95%CI, -13.94 to 13.98; P=0.966) and over time (0.44 mLO_2_·kg^-1^·min^-1^: 95%CI, -13.5 to 12.4; P=0.439). Demographic and anthropometric attributes, cardiovascular risk, and beta-blockers are valuable for predicting *V̇*O_2max_ at baseline and over time. The developed equations may apply to countries with socioeconomic and demographic characteristics such as Brazil and the USA.

## Introduction

Cardiorespiratory fitness (CRF) is associated with lower cardiovascular (CV) risk and all-cause mortality. On the other hand, low CRF is associated with diabetes mellitus, arterial hypertension, coronary artery disease, and several types of cancer (1). CRF has similar predictive power for cardiovascular events as the combined classic cardiovascular risk factors (CVRFs) (1). For a given increase of one metabolic equivalent (MET) in CRF there is a 10 to 30% decrease in the risk of death (2). Considering that most people in the general population can achieve a 1 MET increase in CRF (i.e., 3.5 mLO_2_·kg^-1^·min^-1^ in *V̇*O_2max_), improving CRF should be considered a primary strategy to reduce the risk of chronic disease and improve outcomes across the health spectrum. Accordingly, CRF should be considered a vital sign for health screening in clinical practice (1). When maximal exercise testing is not possible, estimation of *V̇*O_2max_ through non-exercise variables is a valid alternative when resources are limited (3-6).

Although these prediction models have clinical relevance, some gaps should be addressed to enable a more precise estimation of CRF. With rare exceptions, *V̇*O_2max_ prediction equations are based on demographic and anthropometric variables, without considering classic CVRFs (7). Although they are valuable for diagnosing exercise intolerance and its causes (8), they do not allow longitudinal follow-up of CRF considering changes in CVRFs. The changes in CRF levels throughout one's lifespan indicate that a single measurement is insufficient for accurately predicting long-term health outcomes (9). Individuals who are unfit and become fit demonstrate a substantial risk reduction of all-cause mortality compared to those who remain unfit (i.e., up to 44%) (10) and the risk decreased even more in those who remain fit over time (i.e., up to 61%) (11). Therefore, there is ample evidence that changes in CRF over time, whether increasing or decreasing, are associated with substantial reciprocal changes in mortality risk (9). Also, all but one available equation were developed in cross-sectional studies, without considering the individual random variation in *V̇*O_2max_ over time; this longitudinal equation was developed by indirect estimation of *V̇*O_2max_ using the final treadmill workload (12). Finally, we are unaware of studies that developed directly-measured *V̇*O_2max_ prediction in a longitudinal design and combined data from developed and developing countries. *V̇*O_2max_, a measure of aerobic capacity, can be improved through regular physical activity and training, with improvements ranging from 10 to 30%. However, genetic factors play a significant role, accounting for up to 25 to 50% of the variation in *V̇*O_2max_ values (13). Studies have shown differences in *V̇*O_2max_ values between international and Brazilian data (14,15). Accordingly, we aimed to develop reference equations to predict directly measured *V̇*O_2max_ over time based on non-exercise variables in adults undergoing cardiopulmonary exercise testing (CPET) using a treadmill as the exercise mode.

## Material and Methods

The procedures used to acquire and manage data for the FRIEND registry (Fitness Registry and the Importance of Exercise National Database) have been previously reported (16). In summary, laboratories that, in the opinion of the advisory board, use valid and reliable calibration and CPET procedures administered by experienced personnel, were invited to join the FRIEND Registry. Those laboratories that performed CPET contributed data collected between April 2014 and May 2022 to the FRIEND registry. The laboratory on the Brazilian side was invited to contribute to CPETs carried out in its research projects, mostly from projects funded by the Young Researcher Research Project of the São Paulo Research Foundation (FAPESP).

All the data included in the present study that were contributed to the FRIEND Registry were collected by laboratories conducting research for which informed consent was obtained from all individuals submitted to CPETs.

### Cohort

We analyzed treadmill tests performed by 2,170 adults (1,307 men; 20-85 years) from the United States (n=1,880) and Brazil (n=290) with a second valid test after 0.5 to 40.5 years. For participants with more than two tests in the databases, we used the baseline and the test with the longest follow-up time for the present study. Participant screening was specific to each laboratory's procedures to rule out contraindications for exercise testing and risk stratification. For this cohort, laboratories provided data on individuals who, at the time of the test, had no known relevant cardiovascular diseases or chronic obstructive pulmonary disease. Inclusion criteria were age >20 years, maximal exercise test performed on a treadmill, and peak respiratory exchange ratio (RER) ≥1.00. Any tests that were terminated due to abnormal clinical findings before achieving maximal voluntary effort were excluded. Equipment calibration was performed according to the manufacturer's specifications before each test and the procedures were carried out by qualified and experienced personnel.

In addition to the primary outcome of the present study, i.e., *V̇*O_2max_, the following variables were collected similarly in the two countries involved: 1) sociodemographic data: age, sex, country, ethnicity, marital status, occupation, and education; 2) Cardiovascular risk factors: arterial hypertension, diabetes mellitus, dyslipidemia, family history of coronary disease, smoking history, insufficient physical activity, and use of beta-blockers, all obtained by self-report. We asked participants whether they performed at least 150 min per week of moderate to vigorous physical activity or at least 75 min per week of vigorous physical activity. We exemplified moderate activities as walking, low-intensity exercise, and weightlifting, and vigorous activities as jogging, running, swimming, cycling, and other aerobic exercises. When participants achieved lower levels of physical activity than those cited above that are related to relevant health benefits, we classified these participants as insufficiently active (17,18); 3) Anthropometry: body mass, height, and body mass index (BMI) (19); and 4) Physiological variables: blood pressure and resting heart rate. We obtained all anthropometric and physiological measurements directly.

### Statistical analysis

We used 90% of the present study's sample to develop the longitudinal equations and the remaining 10% was used for cross-validation of the best model, applying the developed equation, and evaluating its reliability. The participant selection for each group was made randomly using the selection of random cases from the statistical package (SPSS 23.0 statistical software, IBM, USA) using a table of casual numbers.

Unpaired *t*-test and chi-squared test were used to compare differences in the mean values and proportions of the studied variables between men and women and between Brazilians and Americans.

The longitudinal prediction equations for *V̇*O_2max_ were developed using repeated measures linear mixed models. We considered the follow-up as a fixed factor in this model and the individual's identification number as a random factor. Covariates were evaluated by univariate sensitive analysis to identify those with greater correlations with *V̇*O_2max_. Dichotomous variables were coded as 1 for presence and 0 for absence (e.g., presence of arterial hypertension=1; absence=0). Brazil was coded as a factor=0 and the United States as a factor=1. Then, we developed an equation considering the most significant predictors. We adjusted the equation for the follow-up time and the individual random variation of *V̇*O_2max_. Several different models were fitted. At first, the main predictors and the use of beta-blockers were included. Secondly, models included demographic, anthropometric, and cardiovascular risk variables (excluding beta-blockers).

We chose the best model as the one with the combination of a high R^2^ and clinically relevant and easy-to-obtain significant predictors including demographic, anthropometric, CVRFs, and use of beta-blockers.

The reliability of the best equation proposed in the present study was evaluated in the cross-validation sample by calculating the coefficient of variation (CV), intraclass correlation coefficient (ICC) and its 95% confidence interval (95%CI), and the mean difference and 95%CI of the Bland and Altman plot method (20). The SPSS 23.0 statistical software (IBM) was used for all analyses. The probability of alpha error was set at 5%.

## Results

The 2,170 adults aged 20 to 85 years were, on average, overweight (Table 1). Men had a higher prevalence of CVRFs, including higher rates of arterial hypertension, hyperlipidemia, and diabetes mellitus, and use of beta blockers (Table 2).

**Table 1:** General characteristics of the Fitness Registry and the Importance of Exercise National Database (FRIEND) subjects at baseline for the treadmill analysis using an inclusion criterion of respiratory exchange ratio (RER) ≥1.0.

	Age groups (years)						
	20-29	30-39	40-49	50-59	60-69	>70	Total
Men	n=163	n=321	n=391	n=263	n=146	n=23	n=1307
Age (years)	24.0±2.9	34.7±2.8	44.3±2.7	54.0±2.7	63.0±2.6	72.9±2.1	44.0±2.4
Weight (kg)	83.0±19.5	84.5±18.0	85.5±14.9	83.6±14.5	82.2±15.1	85.5±11.6	84.2±16.2
Height (cm)	177.0±9.4	176.8±8.8	177.4±8.2	174.6±2.7	172.1±10.3	175.7±8.4	176.0±9.4
BMI (kg/m^2^)	26.3±5.3	26.9±5.0	27.1±4.0	27.4±4.1	27.7±4.6	27.0±2.8	27.1±4.5
*V̇*O_2max_ (mLO_2_·kg^-1^·min^-1^)	43.8±10.4	40.2±9.3	35.8±8.4	30.8±8.1	27.8±7.6	22.8±5.3	35.8±10.2
Peak HR (beat/min)	188.9±14.2	189.4±9.1	180.4±12.8	166.8±16.5	155.2±19.0	134.1±20.3	174.5±18.9
Peak RER	1.18±0.16	1.17±0.08	1.17±0.09	1.16±0.09	1.12±0.10	1.14±0.10	1.16±0.09
Women	n=152	n=197	n=231	n=179	n=83	n=22	n=864
Age (years)	23.7±2.8	34.7±2.7	44.2±2.8	54.0±2.7	63.3±2.4	74.3±3.8	43.1±13.3
Weight (kg)	68.1±14.2	70.2±17.4	74.3±16.5	70.1±13.1	75.0±15.0	73.0±17.0	71.4±15.7
Height (cm)	168.3±8.6	166.9±7.1	165.4±7.1	164.3±6.5	163.6±6.1	163.9±7.2	165.8±7.3
BMI (kg/m^2^)	23.9±4.2	25.0±5.5	27.1±5.9	25.9±4.5	28.0±5.7	27.0±5.5	25.9±5.4
*V̇*O_2max_ (mLO_2_·kg^-1^·min^-1^)	40.2±8.7	32.5±7.9	28.9±7.5	26.7±6.5	22.3±5.6	20.5±5.3	30.4±9.2
Peak HR (beat/min)	188.3±10.7	181.3±13.8	178.9±12.4	169.3±14.2	154.4±16.6	145.6±17.3	174.1±17.0
Peak RER	1.15±0.11	1.17±0.09	1.16±0.10	1.16±0.10	1.12±0.08	1.15±0.09	1.16±0.10

BMI: body mass index; *V̇*O_2max_: maximum oxygen uptake; HR: heart rate. Data are reported as means±SD.

**Table 2:** Follow-up length, sex, and percentages of classic cardiovascular risk factors according to country of origin in the Fitness Registry and the Importance of Exercise National Database (FRIEND) cohort.

	Brazil (n=290)	United States (n=1280)
Age (years)	44±14	44±13
Sex (%)		
Males	57.8	60.7
Females	42.8	39.3
Weight (kg)*	74.9±16.7	79.7±16.2
Height (cm)*	165.0±10.5	173.0±9.4
Body mass index (kg/m^2^)*	27.4±5.4	26.5±4.8
Maximum oxygen uptake (mLO_2_·kg^-1^·min^-1^)*	32.3±10.9	33.8±10.0
Presence of cardiovascular risk factors (%)		
Family history of cardiovascular disease*	30.6	10.9
Arterial hypertension*	16.0	30.1
Hyperlipidemia*	26.7	63.8
Diabetes*	9.1	4.8
Current smoking	9.0	7.9
Insufficiently active*	41.7	52.9
Use of beta blockers*	2.1	6.1

The median follow-up time between the first and second exercise test was two years (interquartile range, 0.7 to 7.0; 95%CI, 0.5 to 22.0). *P<0.05: Brazil *vs* United States. Comparison of continuous measures: Student’s *t*-test. Comparison of categorical measures: chi-squared test.

**Figure 1:**
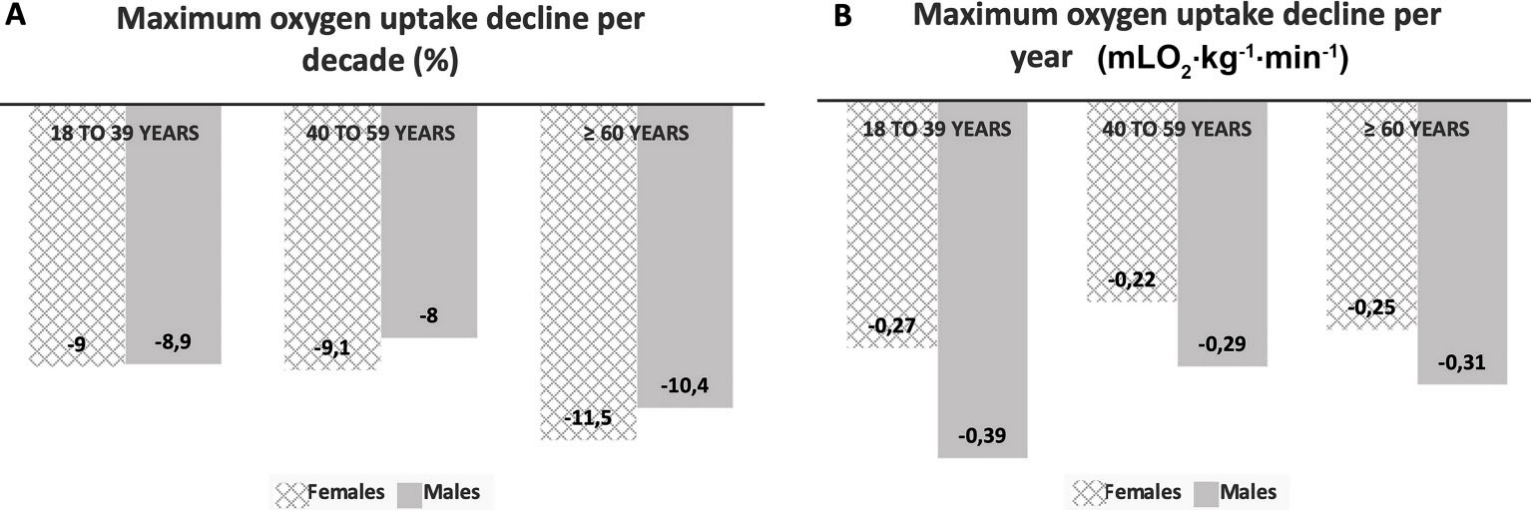
Longitudinal age- and sex-related declines in maximum oxygen uptake: **A**, Absolute values in mLO_2_·kg^-1^·min^-1^ per year and **B**, percent decline per decade.

Results from CPET indicated adequate effort during the CPET (Table 1). Mean maximum oxygen uptake was 5 mLO_2_·kg^-1^·min^-1^ higher for men. The follow-up time of Brazilians was 0.96±1.15 years while of Americans, it was 2.32±5.75 years. Overall, the decline in *V̇*O_2max_ was a median of -0.30 mLO_2_·kg^-1^·min^-1^ per year (interquartile range, -0.71 to -0.31), representing -0.90% per year (-2.03 to 0.98). We found no interaction between age and sex in *V̇*O_2max_ decline, with approximately a 9% decline per decade (Figure 1).

Depending on the inclusion of the use of beta-blockers in the equations, Americans presented more than 2 mLO_2_·kg^-1^·min^-1^ higher *V̇*O_2max_ compared to Brazilians. Brazilians and Americans were heterogeneous in terms of CVRFs (Table 2), justifying the inclusion of the country in the equations. We presented the follow-up time in the total sample as median, interquartile range, and 95%CI, as data was skewed (Table 2).

All equations incorporated *V̇*O_2max_ and all independent variables, including baseline values, and accounted for changes observed over a two-year follow-up period (interquartile range: 0.7 to 7.0; 95%CI: 0.5 to 22.0). Subsequently, we adjusted for follow-up time in the mixed models as a fixed factor. Consequently, the predictions of *V̇*O_2max_ already incorporate the influence of time on repeated estimates of *V̇*O_2max_.

Age, sex, BMI, arterial hypertension, hyperlipidemia, smoking status, insufficient physical activity, country, and use of beta-blockers were the most significant determinants for *V̇*O_2max_. The best equation developed was as follows: *V̇*O_2max_ (mLO_2_·kg^-1^·min^-1^) = 62.013 - (0.230×Age_years_) - (0.001×Age×Age) - (0.653×Body mass index_kg/m_^2^) + (5.471×Sex_females=0; males=1_) + (2.784×Country_Brazil=0; USA=1_) - (0.688×Arterial hypertension_no=0; yes=1_) - (0.453×Hyperlipidemia_no=0; yes=1_) - (2.023×Smoking_no=0; yes=1_) - (4.362×Insufficiently active_no=0; yes=1_) - (1.673×Beta-blockers_no=0; yes=1_); R^2^=0.566 (Table 3). We also developed an equation without beta-blockers (Table 4).

**Table 3:** Results of linear mixed models adjusted for the main predictors, random effects (subjects), and time variables in 90% of the participants from the Fitness Registry and the Importance of Exercise National Database (FRIEND) cohort.

Predictors	Coefficient	Standard error	β	P	[95%CI]	
					Lower limit	Upper limit
Constant	62.01	1.63	37.97	0.000	58.81	65.21
Age (years)	-0.23	0.06	-3.58	0.000	-0.35	-0.10
Age^2^ (years)	-0.001	0.000	-1.92	0.055	-0.002	0.000
Sex	5.47	0.33	16.36	0.000	4.81	6.12
BMI (kg/m^2^)	-0.65	0.03	-21.06	0.000	-0.71	-0.59
Country	2.78	0.43	6.46	0.000	1.93	3.62
Arterial hypertension*	-0.68	0.31	-2.17	0.030	-1.31	-0.06
Hyperlipidemia*	-0.45	0.25	-1.75	0.080	-0.96	0.05
Current smoking*	-2.02	0.51	-3.96	0.000	-3.02	-1.02
Insufficiently active*	-4.36	0.22	-19.32	0.000	-4.80	-3.91
Use of beta-blockers*	-1.67	0.63	-2.62	0.009	-2.92	-0.42

BMI: body mass index; Sex: males=1 and females=0; Country: United States=1 and Brazil=0. *Risk factor: yes=1; no=0. R^2^: within=0.3516; between=0.5901; overall=0.5663. Maximum oxygen uptake can be estimated as follows: *V̇*O_2max_ = 62.01 - (0.23×Age) - (0.001×Age×Age) + (5.47×Sex) - (0.65×BMI) + (2.78×Country) - (0.68×Arterial hypertension) - (0.45×Hyperlipidemia) - (2.02×Current smoking) - (4.36×Insufficient physical activity) - (1.67×Use of beta-blockers).

**Table 4:** Results of the linear mixed models adjusted for the significant predictors, not including the use of beta blocker medications, random effects (subjects), and time variables in 90% of the participants from the Fitness Registry and the Importance of Exercise National Database (FRIEND) cohort.

Predictors	Coefficient	Standard error	β	P	[95%CI]	
					Lower limit	Upper limit
Constant	61.74	1.62	37.95	0.000	58.55	64.93
Age (years)	-0.20	0.06	-3.23	0.001	-0.33	-0.08
Age^2^ (years)	-0.001	0.000	-2.43	0.015	-0.002	-0.000
Sex	5.60	0.33	16.86	0.000	4.95	6.25
BMI (kg/m^2^)	-0.66	0.03	-21.42	0.000	-0.72	-0.60
Country	2.69	0.42	6.37	0.000	1.86	3.52
Arterial hypertension*	-0.94	0.30	-3.07	0.002	-1.55	-0.34
Current smoking*	-2.01	0.50	-3.99	0.000	-3.00	-1.02
Insufficiently active*	-4.47	0.22	-19.94	0.000	-4.911	-4.03

BMI: body mass index; Sex: males=1 and females=0; Country: United States=1 and Brazil=0. *Risk factor: yes=1; no=0. R^2^: within=0.3499; between=0.5920; overall=0.5683. Maximum oxygen uptake can be estimated as follows: *V̇*O_2max_ = 61.74 - (0.20×Age) - (0.001×Age×Age) + (5.60×Sex) - (0.66×BMI) + (2.69×Country) - (0.94×Arterial hypertension) - (2.01×Current smoking) - (4.47×Insufficient physical activity).

**Figure 2:**
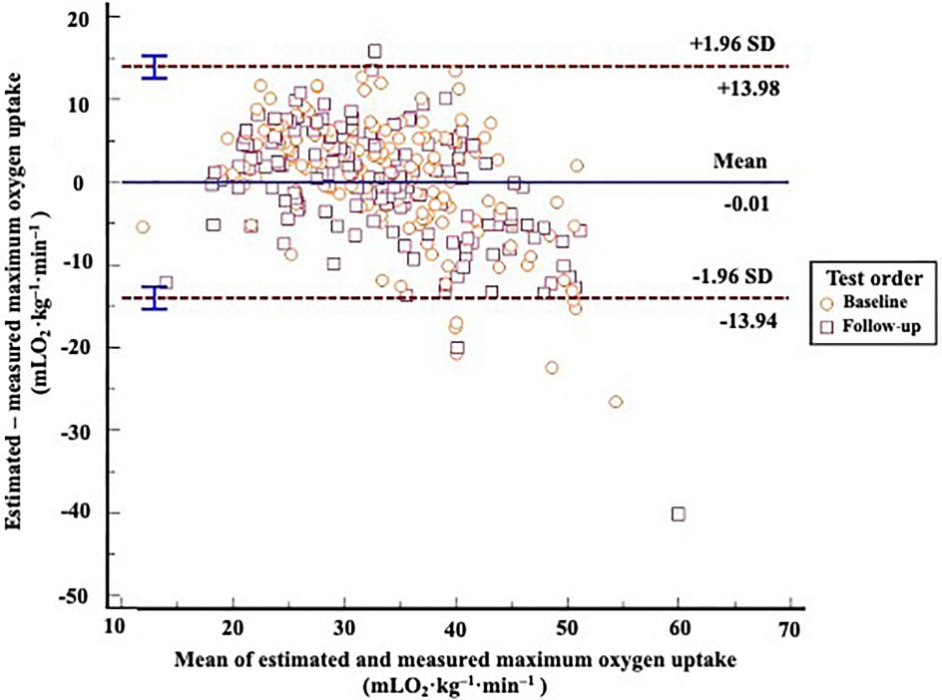
Bland and Altman plot with the limits of agreement between estimated and measured maximum oxygen uptake (*V̇*O_2max_) in 10% of the participants from the Fitness Registry and the Importance of Exercise National Database (FRIEND) cohort. The mean difference was non-significant (P=0.966). The slope of the regression line was -0.036 (P<0.05).

The difference and 95%CI between measured and estimated *V̇*O_2max_ in the cross-validation sample using our main equation (Table 4) was small (0.01 mLO_2_·kg^-1^·min^-1^:-13.94 to 13.98; P=0.966) (Figure 2). Our equation was also reliable over time (difference in *V̇*O_2max_ after follow-up =(0.44 mLO_2_·kg^-1^·min^-1^: -13.5 to 12.4; P=0.43). Overall, the ICC between measured and estimated *V̇*O_2max_ was 0.878 (95%CI: 0.829 to 0.913) and CV was 14%.

## Discussion

We developed treadmill *V̇*O_2max_ prediction equations based on longitudinal data from the FRIEND Registry. To our knowledge, this is one of the first studies of CRF estimation in a longitudinal design measured through gas analysis. Also, we developed equations with data from participants in both a developed and a developing country.

Several reference equations for *V̇*O_2max_ have been published in the literature in the United States (21-23) and Brazil (7,24). As in the present study, some of these equations were developed using the FRIEND Registry. Dourado et al. (25) used the equation developed in Table 4 and observed that our equation was more reliable over time than that of de Souza e Silva et al. (26), which was developed in a cross-sectional study (0.44 mLO_2_·kg^-1^·min^-1^: -13.5 to 12.4; P=0.439 *vs* 1.66 mLO_2_·kg^-1^·min^-1^: -14.9 to 18.1; P=0.016). Accordingly, our equation was reliable at baseline and over time and may be applicable in both developed and developing countries.

Cáceres et al. (7) developed equations including CRVFs in a robust sample of Brazilian participants in a cross-sectional design. Peterman et al. (27) investigated the validity of the equation and another 26 *V̇*O_2max_ prediction equations in a longitudinal study with a mean follow-up of 3.2±5.2 years. They compared changes in estimated CRF (eCRF) from 27 distinct non-exercise prediction equations with the changes in directly measured CRF. The changes in eCRF were significantly different from those in directly measured CRF. The median percentage of participants correctly classified as having increased, decreased, or no change in CRF was 56%, with a range of 39 to 61%, indicating limited clinical applicability of these equations over time. Of the 27 equations studied by Peterman et al. (27), only one (12) was developed from longitudinal data with 1,325 women and 10,040 men between 20 and 86 years who completed two treadmill tests in 10 years of follow-up. However, CRF was quantified using the speed and inclination of the final minute of the treadmill test converted to METs under a Balke protocol, which is related to local fatigue in the lower limbs compromising predicted *V̇*O_2max_ (28). Due to its longitudinal design and the *V̇*O_2max_ evaluated directly from the treadmill protocol, the present study might improve *V̇*O_2max_ prediction over time.

We observed a decline of less than 1% in *V̇*O_2max_ per year, around 9% per decade. The absolute change was between -0.22 and -0.39 mLO_2_·kg^-1^·min^-1^ per year. We did not observe a more significant decline in the group of older individuals. Our results were similar to those described in cross-sectional studies (29). Dourado et al. (30) evaluated 1295 Brazilians undergoing CPET on a treadmill and observed declines between 8.7 and 9.4% of *V̇*O_2max_ in men and women, respectively. Letnes et al. (29) reviewed several *V̇*O_2max_ prediction studies, most commonly reporting an absolute change of around -0.3 to -0.5 mLO_2_·kg^-1^·min^-1^ per year and a generally acceptable percent linear decline of about 10% per decade. However, the decline in longitudinal studies is often greater, around 15 to more than 20% per decade (31,32). Unlike cross-sectional studies, longitudinal studies are scarce, with small samples and a narrow age range. The two largest previous studies investigated *V̇*O_2max_ decline over time in longitudinal designs. Fleg et al. (31) submitted 375 women and 435 men to CPET on a treadmill with a follow-up of 7.9 years. They observed a decline of 3 to 6% between 20 and 30 years of age and greater than a 20% decline in participants aged 70 years and over. Similarly, Letnes et al. (32) observed that the decline in *V̇*O_2max_ was non-linear, from about 3% at age 30 to 20% in the eighth decade of life in 1471 participants with over ten years of follow-up. Although the *V̇*O_2max_ decrease in older participants was also higher in the present study, it did not reach statistical significance compared to the other age groups. The discrepancy between cross-sectional and longitudinal studies, especially at older ages, is mainly due to a more compromised health of the elderly in longitudinal studies compared to participants in cross-sectional studies. The elderly in longitudinal studies, especially with longer follow-ups, are many more survivors, i.e., the naturally more compromised cardiorespiratory fitness is probably related to selection and survival bias.

Our best and more clinically relevant equation showed adequate reliability. However, as shown in Figure 2, it underestimated the *V̇*O_2max_ of participants with higher values in the cross-validation sample (i.e., *V̇*O_2max_ >45 mLO_2_·kg^-1^·min^-1^). Our results were similar to the literature when using anthropometric measurements for *V̇*O_2max_ prediction. Baynard et al. (22) also used the FRIEND Registry to develop reference equations using 4030 subjects and validated them in another 1000 subjects. The equations included waist circumference or BMI in addition to age and sex. Similar results were described by Baynard et al. (22) with *V̇*O_2max_ underestimations for individuals with greater cardiorespiratory fitness, especially those with values greater than 50 mLO_2_·kg^-1^·min^-1^.

Although it seems a limitation, we developed equations for use in clinical practice and primary health care. Maximum O_2_ uptake as high as 45-50 mLO_2_·kg^-1^·min^-1^ will rarely be reached in such settings, which makes our equations perfectly applicable as a routine assessment strategy. There is evidence from 37 studies involving more than 2 million participants that each 3.5 mLO_2_·kg^-1^·min^-1^ increase in estimated *V̇*O_2max_ is associated with an approximately 11% reduction in mortality risk and that participants with *V̇*O_2max_ in the upper tertile had 45% lower mortality among more than 110,000 registered deaths (33). Such associations were independent of age, sex, follow-up, assessment method, or year of publication.

The main reasons for not implementing the direct assessment of cardiorespiratory fitness as a routine in clinical practice are the cost of the assessment, the logistics, and the training of human resources. Fortunately, the advancement of technology has made gas analyzers, ergometry ECG systems, and ergometers increasingly cheaper, making this assessment more accessible in settings with fewer resources. Although this could make assessment a reality for developed countries like the USA, it will still be a distant reality for developing countries such as Brazil, despite the advances and lower costs. Therefore, *V̇*O_2max_ estimates will be the most feasible strategy in this context in the indefinite future.

The *V̇*O_2max_ of Americans was significantly higher compared to Brazilians despite the higher proportion of self-reported arterial hypertension, dyslipidemia, insufficient physical activity, and use of beta-blockers. Reference equations for *V̇*O_2max_ in developed countries have been shown to overestimate the CRF of Brazilians (24). The reason for the lower CRF of Brazilians is unknown. Only a few studies investigated the determinants of *V̇*O_2max_ in low- and middle-income countries, and anthropometric and body composition variables are among the main predictors of *V̇*O_2max_ (34). In fact, Americans in the present study were taller and had a lower BMI, which may partially explain the difference (Table 4). Unfortunately, we did not assess fat-free mass, which is one of the main predictors of *V̇*O_2max_ and could explain the country-related difference found. Also, even though Brazil and the USA are different in terms of income and race, we were unable to explore the role of education level, socioeconomic status, and ethnicity on *V̇*O_2max_ (34). Even with the scarcity of studies on *V̇*O_2max_ determinants in low- and middle-income countries, it is possible to speculate that such variables are involved in the differences found between Brazilians and Americans.

The main strength of the present study is that it is one of the few studies with a longitudinal design and has a pioneering role in assessing *V̇*O_2max_ directly through gas analysis on a treadmill. It also involved participants from developed and developing countries, and it may be usable in countries with similar sociodemographic features. Another strong point was the inclusion of classic cardiovascular risk factors and the use of beta blockers in the proposed algorithms. These characteristics enable the use of the equations both for the diagnosis of exercise intolerance and its causes, not considering cardiovascular risk factors, in routine clinical practice, and in primary health care as a cardiovascular risk screening strategy.

The present study also had limitations that should be considered. Although the maximum follow-up was 40 years, the average follow-up was only about two years. We recognize that our findings may not be generalized for long follow-ups. Although some reports have highlighted geographical differences and nonlinear declines, the expected decline in *V̇*O_2max_ is generally accepted to be linear and about 10% per decade (15,29). Our study also involved only one developing country and one developed country. Therefore, the generalization of our results to other populations must be done with caution. Furthermore, although all contributing laboratories have experience in CPET, and maximum effort was guaranteed using gas exchange ratio, data in the FRIEND Registry were collected with different equipment, protocols, human resources, and data collection procedures. However, the FRIEND initiative has strict guidelines for procedures and the quality of data provided to its database, which minimizes information bias. Finally, only Brazilian participants underwent spirometry and presented values compatible with the absence of ventilatory disorders. American participants only self-reported the absence of respiratory diseases. Given the known association between lung function and cardiovascular risk (35), exercise capacity (36), and mortality (37), some participants with unknown respiratory conditions may have been included in the data analysis.

We can conclude that demographic and anthropometric attributes, classic cardiovascular risk factors, and beta-blockers are crucial to predict *V̇*O_2max_ in a longitudinal design, and the equation composed of the most clinically relevant variables was reliable at baseline and over time. Moreover, our equations can be applied to Brazilians and Americans. Although we cannot generalize our results for all developed and developing countries, our equation might be useful in countries with similar socioeconomic and demographic characteristics.
